# Predicting age from resting-state scalp EEG signals with deep convolutional neural networks on TD-brain dataset

**DOI:** 10.3389/fnagi.2022.1019869

**Published:** 2022-12-06

**Authors:** Mariam Khayretdinova, Alexey Shovkun, Vladislav Degtyarev, Andrey Kiryasov, Polina Pshonkovskaya, Ilya Zakharov

**Affiliations:** Brainify.AI, Dover, DE, United States

**Keywords:** human brain, brain aging, resting-state EEG, deep learning, relative loss

## Abstract

**Introduction:**

Brain age prediction has been shown to be clinically relevant, with errors in its prediction associated with various psychiatric and neurological conditions. While the prediction from structural and functional magnetic resonance imaging data has been feasible with high accuracy, whether the same results can be achieved with electroencephalography is unclear.

**Methods:**

The current study aimed to create a new deep learning solution for brain age prediction using raw resting-state scalp EEG. To this end, we utilized the TD-BRAIN dataset, including 1,274 subjects (both healthy controls and individuals with various psychiatric disorders, with a total of 1,335 recording sessions). To achieve the best age prediction, we used data augmentation techniques to increase the diversity of the training set and developed a deep convolutional neural network model.

**Results:**

The model’s training was done with 10-fold cross-subject cross-validation, with the EEG recordings of the subjects used for training not considered to test the model. In training, using the relative rather than the absolute loss function led to a better mean absolute error of 5.96 years in cross-validation. We found that the best performance could be achieved when both eyes-open and eyes-closed states are used simultaneously. The frontocentral electrodes played the most important role in age prediction.

**Discussion:**

The architecture and training method of the proposed deep convolutional neural networks (DCNN) improve state-of-the-art metrics in the age prediction task using raw resting-state EEG data by 13%. Given that brain age prediction might be a potential biomarker of numerous brain diseases, inexpensive and precise EEG-based estimation of brain age will be in demand for clinical practice.

## Introduction

The human aging process occurs on many levels. In particular, the impact of aging on the brain can be observed throughout the human lifespan. The structural connectivity between the hemispheres and functional connectivity (FC) between distinct regions in the brain increase during aging (Madden et al., 2020). FC represents the spatial–temporal correlations between brain networks observed in a task or resting state conditions (Di and Biswal, 2015). At a certain point around 65 years of age, a gradual decline in FC is observed in normal aging (Siman-Tov et al., 2017; Madden et al., 2020). According to Siman-Tov et al. (2017), brain maturation after the age of 65 has a pronounced impact on connection strength across regions, making some connections weaker, which coincides with the early stages of numerous cognitive dysfunctions.

Some people with mental health conditions are more prone to experience neurological and cognitive dysfunctions early on. For instance, there is growing evidence of FC abnormalities in individuals with depression, bipolar disorder, schizophrenia, as well as neurodegenerative conditions (Bresnahan et al., 1999; Oh et al., 2019; Albano et al., 2022; Metzen et al., 2022). For example, major depressive disorder has been linked to a more prevalent and hyper-connected default mode network (Tang et al., 2022; for a meta-analysis, see Kaiser et al., 2015). Critically, recent studies have highlighted variability between chronological age and accelerated brain aging in people with mental disorders and early life stress (Dunlop et al., 2021; Herzberg et al., 2021). The severity of abnormal fluctuations in FC compared to scans of healthy individuals can be used to identify internalized processes, such as abnormal brain aging, that do not match chronological age (Dunlop et al., 2021). These alterations in cortical dynamic properties have been linked to cognitive dysfunctions observed across neuropsychiatric conditions (Dunlop et al., 2021). This finding has led to the hypothesis that the age of the brain may serve as a biomarker to diagnose certain mental and neurodegenerative conditions early on.

Previous research has observed age-related brain changes using various methods, such as electroencephalography (EEG), MRI, functional MRI (fMRI), and positron emission tomography (PET) (Bresnahan et al., 1999; Dimitriadis and Salis, 2017; Zoubi et al., 2018; Dunlop et al., 2021; Rajkumar et al., 2021). It is important to note that while MRI-based methods have high spatial resolution imaging, they lack the temporal precision of EEG. EEG is also by far the safest and most widely available imaging method (Rajkumar et al., 2021). For example, unlike PET, EEG is safe to administer, as it does not include any radiation risks. Additionally, compared with fMRI, EEG is significantly cheaper and easier to use. EEG methods are widely used to record brain activity during the state of rest (rsEEG); cognitive and motor actions, also known as event-related potentials; and sleep. According to Dimitriadis and Salis (2017), reproducible patterns of accelerated brain age can be observed across various frequency bands in resting conditions, indicating the importance of intrinsic brain oscillations.

Much attention has been drawn to low-frequency alternations in FC observed in rsEEG in people with mental health disorders (Metzen et al., 2022). For example, studies in depression have shown abnormal values of FC dynamics in the prefrontal-limbic regions and abnormalities in the alpha power band at rest (Jaworska et al., 2012; Metzen et al., 2022). Therefore, understanding the EEG FC dynamic and capturing the mechanism behind accelerated brain aging in people with mental conditions could potentially shed light on accurate diagnosis, in-time intervention, and early remission onset.

Overall, a limited number of studies have assessed rsEEG recordings to predict brain age (Dimitriadis and Salis, 2017; Zoubi et al. (2018). Both studies relied on quantitative EEG features processed by traditional machine learning algorithms. Zoubi et al. used a general linear model, while Dimitriadis and Salis employed support vector regression to evaluate brain age prediction. However, approaches based on automated feature generation such as deep convolutional neural networks (DCNNs) have shown better results than traditional machine learning.

DCNNs have shown promising results in pattern recognition and computer vision applications (Sharma et al., 2018; Yamashita et al., 2018; Alzubaidi et al., 2021). This is due to their ability to automatically extract significant spatiotemporal features that best represent the data from its raw form without preprocessing or human decisions necessary for selecting these features (Zeiler and Fergus, 2013; Olah et al., 2017). Owing to these properties, convolutional networks have supported advances in solving many medical problems, including the diagnosis of brain tumors by MRI (Çinar and Yildirim, 2020; Irmak, 2021) and lung diseases by X-ray images (Bharati et al., 2020; Singh et al., 2021). They have also been used to solve the image segmentation problem (segmenting non-overlapping image areas that have unique features) of medical images, highlighting experts’ areas of interest (Feng et al., 2020). Recently, DCNNs have been used to identify biomarkers and diagnose mental disorders using computer tomography and MRI images (Vieira et al., 2017; Noor et al., 2020). Finally, deep learning has been successfully used to solve tasks related to predicting mental diseases from resting-state EEG recordings (Oh et al., 2019; Li et al., 2020; Sun et al., 2021; Sundaresan et al., 2021) and to predict the sex of the brain (Van Putten et al., 2018; Bučková et al., 2020). Thus, deep learning is a promising technology for extracting information from a complex data source, such as human brain EEG, without the need for manual feature engineering.

Computer vision researchers frequently face the problem of insufficient data to train deep learning models. Data augmentation is as a typical approach to solving this issue. It has been used in the overwhelming majority of computer vision studies, and it extends the training dataset with synthesized data obtained by applying various transformations to existing samples (Shorten and Khoshgoftaar, 2019). Unfortunately, the majority of deep learning studies involving EEG data disregard this method, which results in the under-performance of the models. It is possible to increase the size of the original EEG dataset with data augmentation by an order of magnitude, endowing the model with the property of generalization and thereby improving its quality.

One issue that has limited progress in this area is that sample sizes of typical EEG studies are relatively small (e.g., N < 100 subjects), especially for machine learning algorithms. To obtain a larger dataset, machine learning researchers sometimes use separate EEG epochs (segments of EEG records 2–5 s long) for analysis. The lack of data also leads to the use of a cross-validation method instead of testing on a separate hold-out dataset. However, this approach increases the risk of using random cross-validation, which can lead to inflated metrics. Since a deep learning model often memorizes the session’s fingerprint and, consequently, the subject, with all the metrics used (e.g., age or diagnosis), a cross-subject cross-validation method would be beneficial in addressing this risk.

In sum, only two studies have harnessed resting-state EEG recordings for age prediction; Zoubi et al. (2018) reported a mean absolute error ($MAE,inyears)\;of\;6.87\;\left(R^{2}=0.37\right),$and Dimitriadis and Salis (2017) showed $R^{2}=0.60\;\left(eyesopen\right),\;\;0.48\;\left(eyesclosed\right).$We believe it is possible to improve these results since neither study used deep learning techniques. Based on recent developments, we propose the following aims: (1) to prove that a DCNN can be effectively used for brain age prediction from resting-state EEG recordings; (2) to exploit deep learning techniques and assess their effects; (3) to use an impartial data-leak-free cross-subject cross-validation method for training and testing on a large-scale Two Decades–Brainclinics Research Archive for Insights in Neurophysiology (TD-BRAIN) database containing more than a thousand EEG sessions (Van Dijk et al., 2022); and (4) to explain what information coming from raw EEG data is essential for the DCNN and investigate its performance.

## Materials and methods

### Dataset

The current study is based on the TD-BRAIN EEG database, which is a clinical lifespan database containing resting-state raw EEG recordings complemented by relevant clinical and demographic data from a heterogeneous collection of psychiatric patients collected between 2001 and 2021 (Van Dijk et al., 2022). An initial dataset consisted of 1,274 patients (620 females), aged 38.67 ± 19.21 (range 5–88) years, with a total of 1,346 EEG sessions. The sample contained both healthy participants (*N* = 47) and patients with major depressive disorder (MDD; *N* = 426), attention deficit hyperactivity disorder (ADHD; *N* = 271), subjective memory complaints (SMC; *N* = 119), and obsessive–compulsive disorder (OCD; *N* = 75). For 70 participants, more than one session recorded at different times were available. The time interval between the repeated sessions was from 2 months to 14 years (mean interval, 1.16 years; the distribution of the intervals is presented in Supplementary Figure 1). Given the considerable time difference between sessions and that participants’ ages changed from session to session, we treated each session individually. For each session, the participant’s metadata included their age at the time of recording. After the removal of sessions with missing metadata and artifact rejection, the final dataset consisted of 1,335 sessions (719 females, aged 5–88 years, with a mean age 38.8 ± 19.1 years) of eyes-open (EO) and eyes-closed (EC) blocks. The preliminary studies showed that the results did not significantly differ between the dataset consisting of only individual recording sessions and the repeated sessions dataset; thus, the latter was used for the final analysis. In addition to raw EEG recordings, the TD-BRAIN database contains autonomic measures such as electro-cardiography (ECG), electromyography (EMG), and electrooculography (EOG), which are used in cleaning artifacts from raw EEG data.

Psychophysiological data included 26-channel (10–10 electrode international system, Ag/AgCl electrodes) EEG recordings with a sampling rate of 500 Hz (low-pass filtered at 100 Hz before digitization) and a skin resistance level kept below 10 kΩ in a standardized EEG laboratory setup. The EEG was referenced offline to averaged mastoids (A1 and A2) with a ground at AFz. Vertical and horizontal eye movements were recorded with electrodes placed 3 mm above the left eyebrow, 1.5 cm below the left bottom eyelid, and 1.5 cm lateral to the outer canthus of each eye, respectively. The EEG data were recorded during the resting state with 2 min of eyes-opened and eyes-closed conditions (4 min in total). During the eyes-open condition, subjects were asked to rest quietly with their eyes open while focusing on a red dot at the center of a computer screen. During the eyes-closed condition, subjects were instructed to close their eyes and sit still.

### EEG signal preprocessing

We utilized established automatic preprocessing (BrainClinics Resources, 2022) to remove noise and other artifacts (e.g., eye blinks or muscle activity) from the raw EEG recordings. First, data were bandpass-filtered between 0.5 and 100 Hz, and the notch frequency of 50 or 60 Hz was removed. Next, bipolar EOG was calculated and extracted from the EEG signal using the method proposed by Gratton et al. (1983). In the final stage, the following artifacts were detected using various algorithms: EMG activity, sharp channel-jumps (up and down), kurtosis, extreme voltage swing, residual eye blinks, extreme correlations, and electrode bridging (Alschuler et al., 2014). If an artifact was found in the EEG recording, then a mark was put on an additional channel, which was used to remove the segment.

In the TD-BRAIN dataset, EEG recordings are 2 min in length, in turn indicating a considerable probability of the appearance of artifacts, especially in the EO state. To obtain a high-quality sample, all records were divided into segments of identical duration with an overlap and step equal to 1 s. At the same time, the segment was removed from the sample if there was information about the presence of artifacts on the channel received at the preprocessing phase. Experimentally, an optimal splitting duration of 5 s was determined, which allowed us to obtain a model of the best quality as well as a significant amount of clean data (198,648 segments) (see the “Optimal segmentation of EEG recordings” section for further details).

### Machine learning analysis: Cross-subject cross-validation

**Figure 1:**
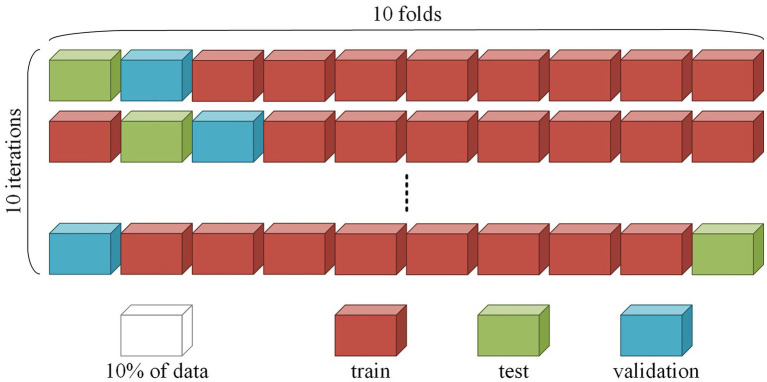
Example of splitting for 10-fold cross-validation.

To correctly assess the model quality, we used 10-fold cross-subject cross-validation with separate validation and testing datasets. The cross-validation procedure was repeated 10 times. At each iteration, the whole dataset was divided into 10 parts, whereby eight parts were used for training the network, one for validation during training, and one for testing the final model. An example of splitting is shown in Figure 1. During training, it was essential to correctly divide the data, since the quality of the model depended on the chosen data split. We placed all EO and EC session segments corresponding to the same subject in the same fold; thus, the model was used to detect patterns among different EEG recordings and not memorize sessions.

### Machine learning analysis: Data augmentation

To increase the training dataset size and improve the model’s quality, we applied the following transformations with experimentally identified parameters to preprocessed EEG recordings as the data augmentation technique:

- with a probability of 50%, apply gaussian noise to the input tensor with random standard deviation drawn from a uniform distribution [0, 1] μV;
- with a probability of 70%, apply random dropout of *B_k_* consequent time-points in k EEG channels of the input tensor data, where *k* and *B_k_* are drawn from uniform distributions [1, 8] and [1, 1800], respectively;
- with a probability of 50%, apply random amplification of the input tensor with a multiplier $M_{ch}$ drawn from a uniform distribution [0.8, 1.2] for each EEG channel ch;
- with a probability of 50%, shrink or stretch the time axis with a factor uniform distribution [0.8, 1.2];
- with a probability of 50%, invert the time flow for all EEG channels.

### Machine learning analysis: Model

**Figure 2:**
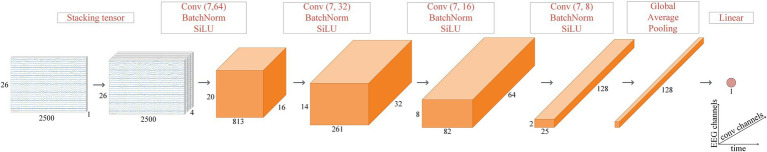
DCNN model structure. The convolutional layers of the central part of the model have stride (1, 3) and the following kernel sizes: (7, 64), (7, 32), (7, 16), and (7, 8). The number of channels changed from 16 to 128, doubling each time. For the EEG segments (first two on the left), the x-axis (time) is in milliseconds.

We used a DCNN with a segment of the EEG recording as an input. The segment was transformed into a stacked tensor (Figure 2) to increase the receptive field of the first convolutional layer. The transformation takes a tensor with dimensions (1, 26, 500*5) as an input for a 26-channel 5-s EEG segment. Then, using a cyclic permutation of channels from top to bottom and concatenating them, a new tensor of dimensions (4, 26, 500*5) was made. The central part of the model is comprised of four blocks, consisting of a convolutional layer, a batch normalization function, and an activation function. The convolutional layer processes the signal with learning weights and resizes the input tensor. The batch normalization technique (Ioffe and Szegedy, 2015) is used to speed up the training of the model and to add regularization by normalizing the data. The sigmoid linear unit is used as an activation function across the convolution layers to add nonlinearity, ensure robustness against noise in the input data, and achieve faster back propagation convergence (Elfwing et al., 2018). After the main blocks, global average pooling is applied to the tensor, transforming the multidimensional tensor into a one-dimensional vector. A linear layer at the end of the model is applied to the vector, whose output is a scalar responsible for the predicted age. Age prediction is performed by applying the model to all artifact-free segments of the EEG session for the eyes-open and eyes-closed tasks, averaged according to Expression 1:


(1) $$Age_{s}=\frac{\sum_{i=1}^{N_{s}}Age_{s}^{i}}{N_{s}}\;$$

where $Age_{s}^{i}\geq 0$ is a predicted age for session $s\in \left\{EO,EC\right\},i=1..N_{s}$, and $N_{s}$ is the number of segments in session s.

### Machine learning analysis: Model training

The main loss function in solving the regression task was Mean Absolute Error, MAE (2). It is suited to the problem of predicting age and is easily interpreted;MAE was used as one of the metrics. The absolute loss function is not always beneficial (see section “Brain age prediction as a classification problem”). Therefore, we applied the mean absolute logarithmic error (MALE), the function that is the ratio of the logarithm of a true value to the predicted one (3). It is less sensitive to the scale of the data and allows for the prediction of smaller values in a more efficient manner.


(2) $$MAE\left(y,\hat{y}\right)=\frac{1}{N}\overset{N}{\underset{i=1}{\sum }}\left|y_{i}-{\hat{y}}_{i}\right|$$

(3) $$MALE\left(y,\hat{y}\right)=\frac{1}{N}\overset{N}{\underset{i=1}{\sum }}|\ln\left(y_{i}+1\right)-\ln\left({\hat{y}}_{i}+1\right)\left|=\frac{1}{N}\overset{N}{\underset{i=1}{\sum }}\frac{y_{i}+1}{{\hat{y}}_{i}+1}\right|,$$

where Nis a sample size, and y and $\hat{y}$ are target and predicted vectors of values, respectively.

During cross-validation, the random partitioning of the sample and the initialization of the weights of the neural network can lead to different values in metrics. Therefore, we used the upper 95% confidence interval ($CI_{95\%}$) of the sample of test metrics from all iterations (4). Some previous studies do not report the MAE metric but do report the coefficient of determination $\left(R^{2}\right)$ metric (5); thus, we also calculated it for a comparison of the results. $R^{2}$ indicates the model fit and is, therefore, an indicator of how well outliers are likely to be predicted by the model through a proportion of the target value variance explained by the model. Thus, using the two metrics together shows not only how the model makes predictions on average but also how well it describes data variance:


(4) $$CI_{95\%}=\bar{x}+\frac{1.96\cdot std}{\sqrt{N_{cv}}}$$

(5) $$R^{2}\left(y,\;\hat{y}\right)=1-\frac{\sum_{i}{\left(y_{i}-{\hat{y}}_{i}\right)}^{2}}{\sum_{i}{\left(y_{i}-\bar{y}\right)}^{2}},$$

where $\bar{x}$ is a mean metric value, std is a metric standard deviation, $\bar{y}=\frac{\sum_{i}y_{i}}{N}$, *N* is a sample size, 1.96 is the approximate value of the 97.5 percentile point of the standard normal distribution, $N_{cv}$ is the number of the cross-validation folds, and the rest of the notation is the same as in Formulas 2 and 3.

The model was trained with pytorch and catalyst (Kolesnikov, 2018) libraries using the Adam optimization algorithm (Kingma and Ba, 2017) with a starting learning rate of $3\cdot 10^{-4}$ and a batch size of 512 segments. As well, we used the “reduce on plateau” scheduler with the patience of three epochs to obtain the maximum quality of the network and the “early stopping” technique after 10 epochs without validation metric improvement to prevent model overfitting. The training was performed on four Nvidia A10G GPUs and took 5 h on average.

## Results

### Age correlations with EEG band power

**Figure 3:**
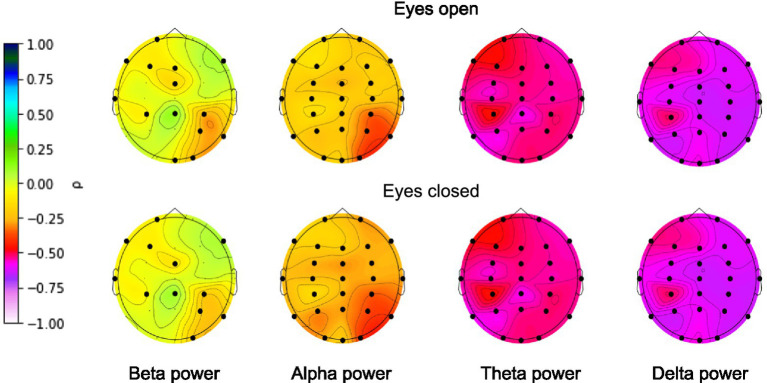
Correlations between age and EEG band power. FDR-corrected significant correlations are marked with a black dot. Color represents the strength of the (non-parametric) Spearman’s correlation coefficient.

To be sure that the EEG signals contained information that could be correctly extracted by the deep learning algorithms prior to brain age prediction, we calculated the zero-order correlations between age and EEG band power (alpha: 8–12 Hz, beta: 12–30 Hz, delta: 1–4 Hz, theta: 4–7 Hz) separately for each EEG electrode. The power of the bands was calculated separately for eyes-closed and eyes-open conditions. The results are presented in Figure 3.

EEG power was shown to be associated with age for all narrow bands for nearly all electrodes. The highest correlations were found for the absolute delta band power and the lowest correlations for the absolute beta band power, with an overall decline in EEG power with age across all bands. The presence of significant correlations allowed us to move on to building the deep learning model.

### Optimal segmentation of EEG recordings

The abundant presence of artifacts in resting-state EEG recordings can deteriorate the quality of the resulting neural network. A frequently used approach is to divide two-minute recordings for eyes-open and eyes-closed states into segments of several seconds, subsequently removing the segments with artifacts from consideration.

With this approach, the task of choosing the optimal duration of one segment arises. As the duration of a segment increases, it becomes easier for the neural network to regress the target variable, as it processes each segment independently of the others. At the same time, deleting a longer segment due to an artifact deprives the neural network of more information compared to a shorter segment. We partially leveled out the latter complexity by using segments intersecting with a step of 1 s. We formulated this task as an optimization problem (6) and (7):


(6) $$SegLen_{optimal}=\left(CrossValMAE\left(\Phi \left(X\right),N_{cv}=10\right)\right)\;$$

(7) X(SegLen)=DataSplit(SegLen,overlap=1),

where $CrossValMAE\left(\Phi ,N_{cv}\right)$ is the cross-validation *MAE* score (in years) calculation for a neural networkΦ with $N_{cv}$ fold iterations; Φ is the neural network function; andDataSplit(SegLen,overlap) is an algorithm splitting records into segments of length SegLen with overlap seconds.

**Figure 4:**
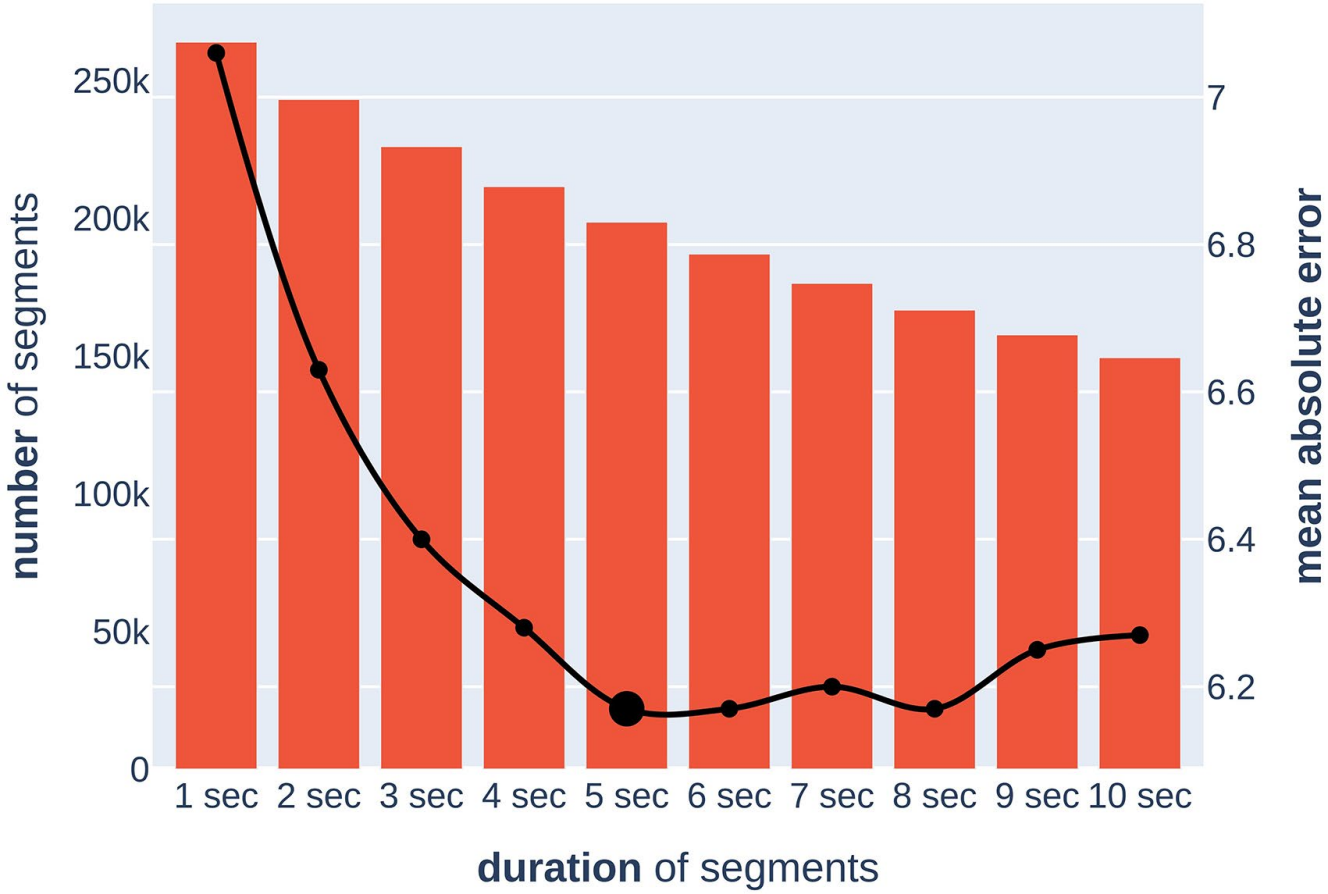
Dependence of model quality on a segment duration (x-axis). The bar chart (left y-axis) shows the number of segments after removing artifacts. The line chart (right y-axis) shows the upper bound of the 95% confidence interval for the MAE (in years) metric of the model.

To solve this problem, we trained 10 independent models on segments of duration from one to 10 s (in the case of 1 s, there was no overlap between segments) and evaluated their quality and the sample size after artifact removal (Figure 4). For reliability, the optimal segment length was chosen based on the upper bound of the 95% MAE confidence interval, which was calculated by cross-validation. The calculated optimal duration of 5 s was used for further experiments. This allowed for the removal of all segments with artifacts while keeping the total number and duration of segments in training at sufficient levels.

Thus, the final prediction of the brain age of a subject was carried out by predicting the age for all five-second artifact-free segments from both EC and EO sessions with subsequent averaging of the obtained values.

### Influence of eye state

We carried out a series of experiments to study the influence of eye state during EEG recording on age prediction. Three DCNNs were trained independently on the following different datasets: only data with open eyes, only data with closed eyes, and data with both conditions. Each of the models predicted these datasets separately (Table 1).

**Table 1:** Performance of models predicting brain age trained for different eye states.

Training data	Testing data: MAE (std; years)		
	Eyes open and closed	Eyes open	Eyes closed
Eyes open and closed	5.96 (0.33)	6.30 (0.37)	6.14 (0.43)
Eyes open	6.44 (0.44)	6.39 (0.37)	7.13 (0.45)
Eyes closed	6.5 (0.47)	7.43 (0.52)	6.33 (0.44)

MAE is in years.

As a result, we observed almost identical single-eye-state model performance on the known modality data [the MAE was 6.39 (for open eyes) and 6.33 (for closed eyes) years]. At the same time, the eyes-closed model experienced more difficulty with the opposite-eye-state data relative to the eyes-open model (MAE = 7.43 years vs. 7.13 years). Thus, the open eyes condition was slightly more informative for the DCNN in predicting brain age than closed eyes. At the same time, the best performance was achieved using both eye states simultaneously. Both modalities acted as additional data augmentations and provided the DCNN with better performance and generalization ability.

### Accuracy of brain age prediction

The results of the present study confirmed the presence of brain age information in the resting-state EEG recordings, which a deep convolutional neural network effectively extracted. To our knowledge, the proposed DCNN architecture predicts human brain age with the best-known quality achieved in the resting-state EEG recordings with MAE = 5.96 (std = 0.33) years and $R^{2}=$0.81 (std = 0.03). All experiments were conducted using robust 10-fold cross-subject cross-validation on a subset of the TD-BRAIN dataset containing resting-state EEG with open and closed eyes.

Table 2 shows the results of the work compared to previous works on the topic. The Pearson correlation coefficient for the samples of true and predicted values was 0.9.

**Table 2:** Metrics of models predicting brain age.

Model	Dataset size, N	Age range and distribution [range, mean ± std, (years)]	MAE [mean ± std, (years)]	$R^{2}$
Dimitriadis and Salis (2017)	194	[18, 67], 37.7 ± 10.2	-	0.60 (EO) 0.48 (EC)
Zoubi et al. (2018)	468	[18, −], 34.8	6.87 ± 0.69	0.37 (EO)
Current model (MAE)	1,335	[5, 88], 38.8 ± 19.1	5.96 ± 0.33	0.81 (EO and EC)
Current model w/o “stacking tensor” and data augmentation	same		6.11 ± 0.50	0.80 (EO and EC)

The table shows the size of the datasets, mean age, and standard deviation (std), as well as the MAE(in years; lower is better) and R^2^ (higher is better) metrics obtained on the corresponding datasets.

The “roll and shift” method and data augmentation played a noticeable role in DCNN quality. The first technique allowed the first layer of the network to obtain more information from the signal, and the second improved the model’s ability to generalize. An increase in the size of the input tensor, and the application of various transformations to the segments of the EEG signal, led to an MAE metric improvement of 2.5% [from 6.11 (std = 0.5) to 5.96 (std = 0.33) years, Table 2]. Applying these methods together seems especially useful, as the network should not only be more precise but also possess better generalization ability.

### Brain age prediction as a classification problem

Although age is a continuous variable, some brain studies consider it to be categorical by dividing participants into age groups (Bresnahan et al., 1999; Gaudreau et al., 2001; Bonnet and Arand, 2007). At the same time, various studies have used different boundaries between groups. The current model makes it possible to find the optimal partition of an entire age range of K non-overlapping groups as follows: let $y=\left(y_{1},\ldots ,y_{N}\right)$ and $\hat{y}=\left({\hat{y}}_{1},\ldots ,{\hat{y}}_{N}\right)$ be the target and predicted age, and $b_{1},\ldots ,b_{k+1}$ are borders for the age groups $C_{1,}\ldots ,C_{k}$ such that $C_{i}=\left[b_{i},\;b_{i+1}\right)$ for $i=\bar{1..K}.$ We will look for boundaries that increase the balanced accuracy score $bAcc\left(Β\left(y\right),Β\left(\hat{y}\right)\right)$ described in Brodersen et al. (2010), where Β(x) is the age matching formula, such as $B\left(x\right)=C_{j}$ if $x\in \left[b_{j},b_{j+1}\right)$. We also set restrictions on the class sizes |C| so that the size of the largest class did not exceed the smallest one by a factor of two such that the classes are more balanced. Thus, the optimization problem of determining the boundaries of age groups has the following form:


(8) $$\begin{array}{l} bAcc\left(Β\left(y\right),Β\left(\hat{y}\right)\right)\to \underset{b_{1}\ldots b_{k+1}}{Argmax}\\ \;\;\;\;\;\;\;\;\;\;\;\;\;\;b_{1}>b_{2}\ldots >b_{k+1}\;\\ \;\;\;\;\;\;\;\underset{i=1\ldots k}{min}\left|C_{i}\right|\geq 0.5\;\underset{i=1\ldots k}{max}\left|C_{i}\right| \end{array}$$

We used the stochastic global search optimization Differential Evolution algorithm (Das and Suganthan, 2011) to solve (8). Table 3 shows the optimal class boundaries found using the mentioned algorithm for K={2,3,4,5}.

**Table 3:** Table of age groups found using the evolutionary algorithm.

K	Age groups boundaries, [*b_i_*, *b*_*i+*1_]	Age group sizes, $C_{i}$	Balanced accuracy, bAcc
2	[5,38),[38,88]	[632,703]	0.897
3	[5,20),[20,46),[46,88]	[281,528,526]	0. 819
4	[5,15),[15,32),[32,52),[52,88]	[209,342,413,371]	0.764
5	[5,18),[18,34),[34,47),[47,56),[56,88]]	[246,290,297,213,289]	0.631

Square brackets indicate that the end of the range is inclusive; parentheses indicate that the end is exclusive.

**Figure 5:**
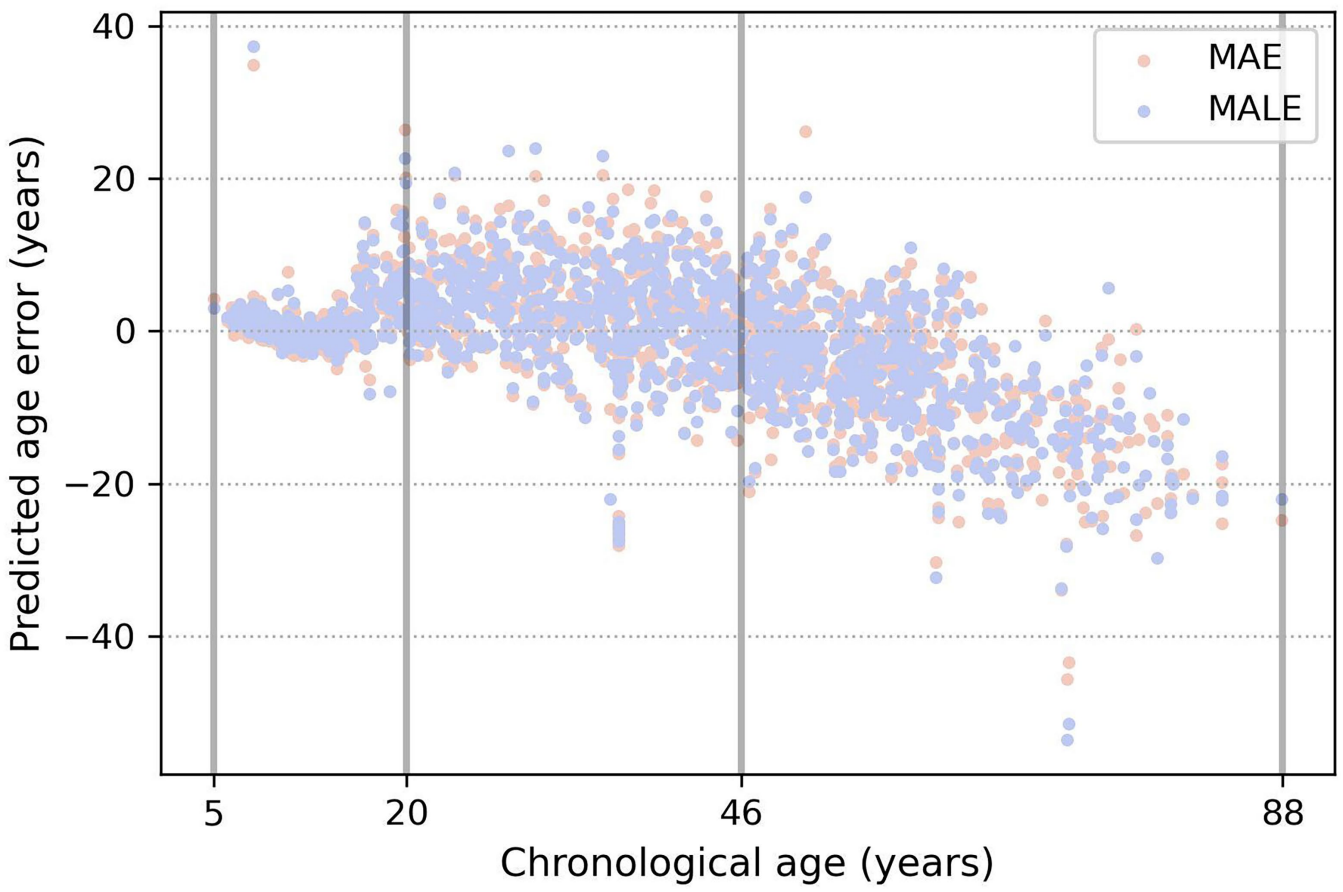
Example of three age groups obtained by the evolutionary algorithm. The true age is marked on the x-axis, and the y-axis shows the difference between the predicted and true age. Orange and blue dots show the prediction errors of the models, trained using the MAE and MALE loss functions, respectively.

From Table 3 and Figure 5, a very prominent young group aged 5–20 is visible: the model predicts it much more accurately than the middle age. There is also a group older than approximately 50 years age, in which the model consistently errs toward a younger age. This seems plausible since the brain develops rapidly at a young age, and a couple of years make a sizable difference, while in old age, a difference of 5–7 years may not be noticeable. These observations led us to conclude that, from a physiological point of view, it would be most natural to optimize not the absolute error of MAE but rather the relative one—for example, MALE.

We trained models with both absolute and relative loss functions and compared their mean absolute error metric in the obtained age groups for K=3. Table 4 shows that DCNN trained with relative loss is more valuable for further application. The metrics indicated that using the MALE loss function reduces the spread of values in the first two age groups, making it possible to predict age more effectively.

**Table 4:** The MAE for the models trained with different loss functions.

Age range	Sample size, N	Absolute loss training [MAE, (years)]	Relative loss training [MAE, (years)]
[5,20]	281	2.45	**2.39**
[20,46]	528	5.78	**5.54**
[46,88]	526	**8.01**	8.34
Full dataset	1,335	**5.96**	5.98

The table shows the found age groups and their size, with the corresponding MAE (in years; lower is better) metrics of two models. Square brackets indicate that the end of the range is inclusive; parentheses indicate that the end of the range is exclusive.

### Importance of cross-subject validation

We noted the critical role of the cross-validation strategy used, since it allows for an objective assessment of the quality of the model. First, the selected number of folds allows for a sufficiently large test set size of more than a hundred sessions. Furthermore, it allows for more accurate estimation of the boundaries of the confidence interval in the resulting metric when compared to a smaller number of folds. Second, cross-subject separation eliminates data leakage. It guarantees the distribution of all information from one session, including open and closed eyes, only inside the training, validation, or testing set. This deprives the neural network of the ability to memorize and use “session fingerprints” for age prediction. The model extracts patterns from the data familiar to different sessions and subjects, ultimately leading to better generalizability. To illustrate the possible data leakage effect, we replaced the cross-subject split rule with a random split. The model trained on 10-fold cross-validation with random mixing of session information between folds achieved MAE=2.03 years and $R^{2}=0.97$. Such metrics look optimistic, but, unfortunately, would not be replicated with new or hold-out EEG sessions.

### Prediction of brain disorders correlated with age

In the present study, the DCNN models are trained and tested on the heterogeneous sample with both health participants and participants with several disorders. To decrease the possibility that the used algorithms identified the probability of a certain disease rather than the age we trained the multiclass DCNN model to predict the TD-BRAIN’s disease status of participant. For the purpose of the analysis, the original indications and formal diagnosis were grouped into 13 classes. Overall, the prediction accuracy of the multiclass model was low. The weighted average prediction accuracy for all classes was 39% (a detailed description of the multiclass prediction analysis can be found in Supplementary Table 1).

### Model explanation

**Figure 6:**
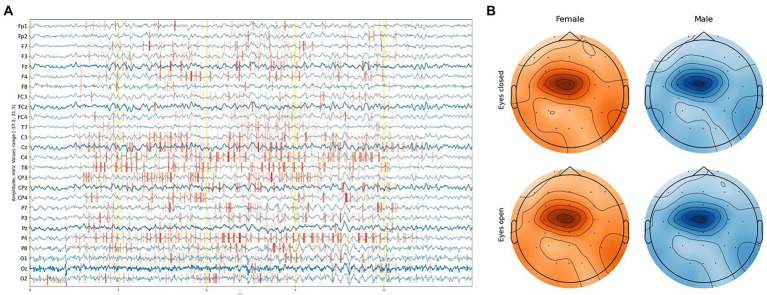
**(A)** Example of an attribution map for one EEG segment. **(B)** Feature importance based on integrated gradient attribution for different sexes and eye states aggregated over all EEG segments.

While DCNNs have had a significant impact on various tasks, explaining their predictions remains a challenging. One approach is to assign an attribution value, also called “relevance” or “contribution,” to each input feature of a network. Given a specific target neuron c, the goal of the attribution method is to determine the contribution $R_{c}=\left[R_{c}^{1}..R_{c}^{N}\right]\in R^{N}$ of each input feature $x_{i}$ to the output $S_{c}$. The problem of finding attributions for deep networks has been tackled in several previous works (Simonyan et al., 2013; Zeiler and Fergus, 2013; Springenberg et al., 2014; Bach et al., 2015; Montavon et al., 2017; Zintgraf et al., 2017). In the examined regression task, there is a single output neuron $S_{c}$ responsible for the age prediction. When the attributions of all input features are arranged to have the exact shape of the input sample, we discuss attribution or sensitivity maps (Figure 6A).

We exploited the Integrated Gradients method proposed by Sundararajan et al. (2017) in conjunction with the Smooth Grad method (Smilkov et al., 2017), which sharpens the sensitivity map. Attribution maps were obtained at a segment level and aggregated along the time dimension, providing a feature importance score with [channel, sex, eye-state] resolution for each segment. The average feature-importance illustration on a topological head map shows its concentration around the Cz channel and slightly to C1 on the left with a slight difference between the eye states and sex of a subject (Figure 6B).

**Figure 7:**
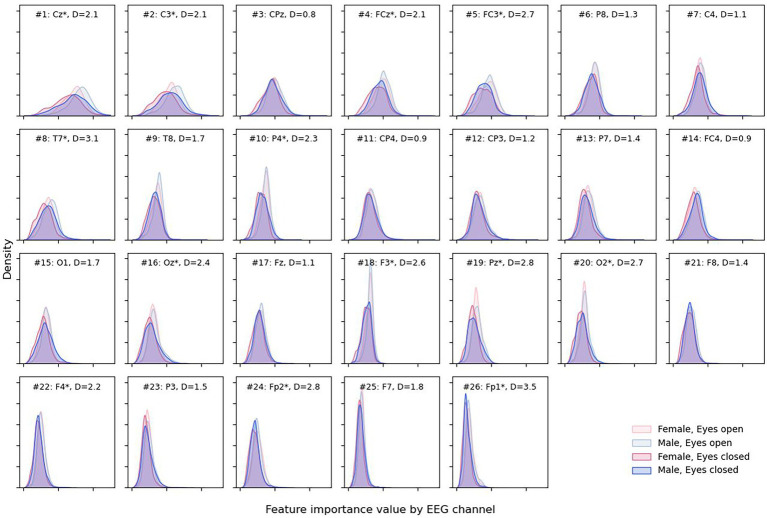
Density plots for male and female sexes and different eye states for each EEG channel. Channels are presented in descending order of total attribution with larger (more interesting) values to the right on the x-axis. Channel is marked with “*” when the absolute difference “D” between medians for eyes open and closed attribution is greater than 2 * IQR for the eyes-closed condition.

More detailed results can be obtained from Figure 7, where almost no difference in the feature importance between sexes can be observed, although with some difference in eye states.

Open eyes shifted to the right, providing slightly more valuable information for the DCNN compared to closed eyes in some but not all channels—Cz, C3, FCz, FC3, etc. The most notable differences between feature importance were found for the Fp1 and Fp2 electrodes (D = 3.5 and D = 2.8, respectively).

## Discussion

In the present study, we aimed to develop a deep learning model for brain age prediction based on the EEG data from the TD-BRAIN dataset (Van Dijk et al., 2022). In line with the existing literature (Anderson and Perone, 2018), the preliminary correlational analysis showed that aging processes are associated with decline in the power of EEG frequency bands, allowing us to train DCNN models. According to our results, brain age information can be extracted from EEG signals with a DCNN with high accuracy when optimal characteristics of the signals and proper data augmentation procedures are used.

### Optimal characteristics for DCNN brain age prediction

In the present study, we used the data augmentation techniques to increase the accuracy of the prediction. While such techniques have been proven successful in the analysis of visual information, in the EEG data, with the network of intercommunicating sources of activity, the implementation of data augmentation may potentially affect the analysis more heavily. However, in the present study, we used the synthetic noise for data augmentation. It was distributed randomly, irrespective of any individual EEG characteristics. The main purpose of the data augmentation is to minimize the distance between the training and test datasets, increasing the variability in the train dataset and helping to solve the overfitting problem (for a more detailed discussion of data augmentation in EEG, see He et al., 2021). To avoid the potential pitfalls related to data augmentation (e.g., false positive or false negative results), we have used the 10-fold cross-subject cross-validation technique. We have also demonstrated a crucial role for correct cross-subject cross-validation—when applied inappropriately, it can lead to serious inflation of the prediction accuracy. The other important result of the study is the introduction of a relative loss function, which works better than the absolute function. According to our results, while the open-eyes condition was slightly more informative for the DCNN in predicting brain age than the eyes-closed condition, for the present task, the best performance was achieved when both eye states were used simultaneously, divided into five-second epochs.

### Accurate brain age prediction from EEG is feasible

This work improves the best-known MAE for brain age prediction based on resting-state EEG by 13% (from 6.82 to 5.97 years), and $R^{2}$ by 35% from (0.60 to 0.81). Our results also indicate that prediction accuracy can differ for different age groups, with the highest accuracy for the participants 15–20 years old. Why was $R^{2}$ increased more than MAE? Presumably, Zoubi et al. (2018) had many outliers and/or their model predicts them poorly. Dimitriadis and Salis (2017), unfortunately, did not report MAE. One important difference between our research and previous work is related to the bigger sample size utilized for the current analysis. It has been recently shown that bigger samples in neuroscience studies are needed for obtaining more stable and reproducible findings (Marek et al., 2022). The improvement in results can be also related to wider age range (the presence of young people under the age of 18) in our dataset. While in our study we achieved prediction accuracy higher than in the rest of the published EEG literature, MRI-based brain age prediction of MAE is significantly higher. In a recent study, Leonardsen and colleagues (Leonardsen et al., 2022) achieved MAE=2.47 years. However, in their study, the deep learning CNN model was trained on a much bigger sample (N = 53,542), leaving the possibility that EEG-based prediction can also be increased with a larger sample. One advantage of EEG brain age prediction compared to MRI brain age prediction is that EEG signals contain high-frequency brain activity, which is crucial for communication within the brain (Fries, 2015). Whether the modality (MRI or EEG) or the sample size is the more important factor in age prediction accuracy is a matter of future studies.

### Brain age prediction as a potential biomarker

The present analysis was done on a heterogeneous dataset consisting of both healthy participants and participants with various disorders. The fact that the disorders are not evenly distributed across the age groups within the dataset leaves open the possibility that the DCNN model could have captured not only information regarding the age of the participant, *per se*, but also the probability of having a particular disease. Although the additional analysis showed that the DCNN models could not accurately predict the type of disease from the same EEG data, the disorders as potential confounders cannot be completely ruled out.

Currently, a promising application of machine learning for age prediction is associated with the delta between prediction from brain characteristics and chronological age (brain-predicted age difference, brain-PAD). The brain-PAD has been previously associated with multiple illnesses. More extreme brain-PAD has been observed in patients with depression (Schmaal et al., 2020), cognitive impairment (Elliott et al., 2021), dementia (Wang et al., 2019), Alzheimer’s disease (Gaser et al., 2013), and schizophrenia (Rokicki et al., 2020). In a recent large-scale MRI study, higher brain-PAD was linked to age-related changes in glucose level, insulin-like growth factor-1, level of glycated hemoglobin, and negative lifestyle habits such as smoking or excessive alcohol consumption (Leonardsen et al., 2022). However, the effect size of the association between MRI-based brain-PAD and various health-related problems was relatively small, suggesting cautious causal interpretation. When compared to EEG, it must be noted that MRI brain-PAD was calculated from structural rather than functional data. While structural and functional brain characteristics are definitely correlated (e.g., white matter connectivity predicts EEG functional connectivity; Chu et al., 2015), the aging processes can affect them differently. This fact can play a crucial role when it comes to correlating brain-PAD with neurological and psychiatric disorders because of their functional rather than anatomical nature (Finn and Constable, 2016). Critically, high-frequency brain oscillations contain information about the dynamic synchronization between different brain areas, forming functional brain networks (Fries, 2015). Alterations within brain networks are now seen as the major source of different disorders (Bassett and Bullmore, 2009; Van Den Heuvel and Fornito, 2014). One way to further increase both the sensitivity and specificity of EEG brain age prediction and brain-PAD as a functional biomarker can be to account for the network information available in EEG synchronization patterns.

### Feature importance and the model explanation

In our study, we have also shown that building activation maps for EEG signals from the DCNN model is feasible. The activation maps have previously shown its utility in the image recognition tasks, including medical image recognition (Hesamian et al., 2019). An advantage of the activation maps as a tool for feature-importance analysis is that it can be used by a neuroscience researcher even without strong data analytical skills. The result of the model explanation in our data showed different results compared to the feature-importance analysis by Zoubi et al. (2018), where the left parieto-temporal area (TP9 electrode according to the 10–10 System) was shown to be the most important factor for age prediction. The difference in most essential regions may be attributed to the difference in approaches—the current study used a DCNN as an automated feature extractor, while the study conducted by Zoubi et al. used a stack-ensemble of classical machine learning algorithms over hand-crafted features. Different machine learning methods can approach the same problem in different ways. Another important aspect to be noted is that while both our analysis and the analysis by Zoubi and colleagues were based on a mix of healthy and clinical samples, the disorders in the two different clinical groups did not match. The generalizability of the results across different samples should be verified in future research on normative EEG and EEG from a broad range of disorders.

The observed higher importance of open eyes rather than closed eyes may be related to the higher vigilance state, activation, and information processing (Barry et al., 2007; Wong et al., 2016). Indeed, we observed a significant difference in the feature importance in the frontal regions, potentially associated with eye movements and eye state. It should be noted that closed versus open eye conditions are accompanied with an overall change in the EEG frequency spectrum, most notably in the frontal areas. The individual differences in the eyes-closed/eyes-open spectral changes can be associated with multiple reasons, e.g., sleep-related problems. Overall, the model explanation analysis showed that the activation maps can be used in addition to more widespread methods that estimate feature importance for deep learning models. The detailed analysis of the neurophysiological characteristics of age-related EEG sections, highlighted by the activation maps method, and its comparison to the results of other methods should be addressed in future research.

### Further work and limitations

Overall, in our study, we have shown that high-accuracy prediction is feasible with resting-state EEG. We believe this to be an important improvement due to the much higher availability and lower cost of EEG technologies. Given that brain-PAD is seen as an important potential biomarker of numerous neurological and psychiatric conditions its inexpensive and precise EEG-based estimation likely to be in demand for clinical practice in areas such as automatic diagnostics and treatment predictions. For example, such projects have now been developed for depression studies (Zhang et al., 2020). It would be reasonable to conduct further research in several directions as follows: first, identifying factors that allow DCNNs to determine the age of the human brain, studying these factors, and verifying them from a neurophysiological point of view; second, creating a neural network with a high generalizability, making it possible to predict the age of the human brain using data collected in new conditions (different site, different equipment, etc.); third, exploring whether there would be benefits to using EEG-informed fMRI (i.e., combining EEG with higher spatial resolution fMRI data). Finally, the model is trained to predict age, but it can also be valuable for transferring identified features from one domain (age prediction in the current study) to another domain (neuropsychiatric disorders). This could allow for the identification of new brain-state biomarkers and the prediction of treatment outcomes for mental disorders.

An important limitation of the current study is the specific dataset used. The current deep learning model was built on EEG data predominantly from patients with different disorders. The accuracy of the prediction must be verified from normative EEG, as well as EEG from people with different types of disorders to ensure the generalizability of the obtained results. However, to our knowledge, the large-scale, normative resting-state EEG of a wide age range has not yet been conducted. Moreover, existing datasets are mostly limited to participants of European ancestry. Creating a large-scale open dataset with a diverse sample is a necessary step for the further development of EEG brain age prediction models. Another limitation relates to the interpretability of the obtained deep learning model. In the present study, we showed the feasibility of an activation map approach to finding the exact features that deep learning models use for brain age prediction. However, the nature of these features was beyond the scope of the current study. We plan to address the neurophysiological properties of activation maps in future research.

## Conclusion

To sum up, according to our results, the deep convolutional neural networks can show higher accuracy in brain age prediction using resting-state EEG signals than other approaches. The DCNN with the introduced loss function outperforms previously used methods by 13% if suitable data augmentation techniques and proper cross-validation procedures for avoiding inflated prediction accuracy are applied. However, in our study, we trained the DCNN on a heterogeneous sample with both healthy participants and participants with different disorders. To ensure the generalizability of the obtained results, the brain age prediction accuracy must be verified in larger and more diverse samples in future research.

## Data availability statement

Publicly available datasets were analyzed in this study. This data can be found at: https://www.nature.com/articles/s41597-022-01409-z.

## Ethics statement

The studies involving human participants were reviewed and approved by data collection sites (Nijmegen: Commissie Mensgebonden Onderzoek for initial data collection, Regio Arnhem-Nijmegen; CMO-nr: 2002/008). Written informed consent to participate in this study was provided by the participants’ legal guardian/next of kin.

## Author contributions

All authors listed have made a substantial, direct, and intellectual contribution to the work and approved it for publication.

## Funding

The research was funded by the Brainfy.AI company in which all the authors are employees.

## Conflict of interest

All the authors were employed by Brainfy.AI.

The authors declare that this study received funding from Brainify.AI. The funder was involved in the study design, analysis, interpretation of data, the writing of this article and the decision to submit it for publication.

## Publisher’s note

All claims expressed in this article are solely those of the authors and do not necessarily represent those of their affiliated organizations, or those of the publisher, the editors and the reviewers. Any product that may be evaluated in this article, or claim that may be made by its manufacturer, is not guaranteed or endorsed by the publisher.

## References

[ref1] Albano L. Agosta F. Basaia S. Cividini C. Stojkovic T. Sarasso E.. (2022). Functional connectivity in Parkinson’s disease candidates for deep brain stimulation. NPJ Parkinsons Dis. 8:4. doi: 10.1038/s41531-021-00268-6, PMID: 35013326 PMC8748462

[ref2] Alschuler D. M. Tenke C. E. Bruder G. E. Kayser J. (2014). Identifying electrode bridging from electrical distance distributions: a survey of publicly-available EEG data using a new method. Clin. Neurophysiol. 125, 484–490. doi: 10.1016/j.clinph.2013.08.024, PMID: 24095153 PMC3943722

[ref3] Alzubaidi L. Zhang J. Humaidi A. J. Al-Dujaili A. Duan Y. Al-Shamma O.. (2021). Review of deep learning: concepts, CNN architectures, challenges, applications, future directions. J. Big Data 8:53. doi: 10.1186/s40537-021-00444-8, PMID: 33816053 PMC8010506

[ref4] Anderson A. J. Perone S. (2018). Developmental change in the resting state electroencephalogram: insights into cognition and the brain. Brain Cogn. 126, 40–52. doi: 10.1016/j.bandc.2018.08.001, PMID: 30144749

[ref5] Bach S. Binder A. Montavon G. Klauschen F. Müller K.-R. Samek W. (2015). On pixel-wise explanations for non-linear classifier decisions by layer-wise relevance propagation. PLoS One 10:e0130140. doi: 10.1371/journal.pone.0130140, PMID: 26161953 PMC4498753

[ref6] Barry R. J. Clarke A. R. Johnstone S. J. Magee C. A. Rushby J. A. (2007). EEG differences between eyes-closed and eyes-open resting conditions. Clin. Neurophysiol. 118, 2765–2773. doi: 10.1016/j.clinph.2007.07.028 17911042

[ref7] Bassett D. S. Bullmore E. T. (2009). Human brain networks in health and disease. Curr. Opin. Neurol. 22, 340–347. doi: 10.1097/wco.0b013e32832d93dd, PMID: 19494774 PMC2902726

[ref8] Bharati S. Podder P. Mondal M. R. H. (2020). Hybrid deep learning for detecting lung diseases from X-ray images. Inform. Med. Unlocked 20:100391. doi: 10.1016/j.imu.2020.100391, PMID: 32835077 PMC7341954

[ref9] Bonnet M. H. Arand D. L. (2007). EEG arousal norms by age. J. Clin. Sleep Med. 3, 271–274. doi: 10.5664/jcsm.26796 17561594 PMC2564772

[ref10] BrainClinics Resources (2022). https://brainclinics.com/resources/

[ref11] Bresnahan S. M. Anderson J. W. Barry R. J. (1999). Age-related changes in quantitative EEG in attention- deficit/hyperactivity disorder. Biol. Psychiatry 46, 1690–1697. doi: 10.1016/s0006-3223(99)00042-6, PMID: 10624551

[ref12] Brodersen K. H. Ong C. S. Stephan K. E. Buhmann J. M. (2010). “The balanced accuracy and its posterior distribution.” in *2010 20th Int Conf Pattern Recognit*. 3121–3124.

[ref13] Bučková B. Brunovský M. Bareš M. Hlinka J. (2020). Predicting sex from EEG: validity and generalizability of deep-learning-based interpretable classifier. Front. Neurosci. 14:589303. doi: 10.3389/fnins.2020.589303, PMID: 33192274 PMC7652844

[ref14] Chu C. J. Tanaka N. Diaz J. Edlow B. L. Wu O. Hämäläinen M.. (2015). EEG functional connectivity is partially predicted by underlying white matter connectivity. NeuroImage 108, 23–33. doi: 10.1016/j.neuroimage.2014.12.033, PMID: 25534110 PMC4323839

[ref15] Çinar A. Yildirim M. (2020). Detection of tumors on brain MRI images using the hybrid convolutional neural network architecture. Med. Hypotheses 139:109684. doi: 10.1016/j.mehy.2020.109684, PMID: 32240877

[ref16] Das S. Suganthan P. N. (2011). Differential evolution: a survey of the state-of-the-art. IEEE Trans. Evol. Comput. 15, 4–31. doi: 10.1109/tevc.2010.2059031

[ref17] Di X. Biswal B. B. (2015). Dynamic brain functional connectivity modulated by resting-state networks. Brain Struct. Funct. 220, 37–46. doi: 10.1007/s00429-013-0634-3, PMID: 25713839 PMC3980132

[ref18] Dimitriadis S. I. Salis C. I. (2017). Mining time-resolved functional brain graphs to an EEG-based chronnectomic brain aged index (CBAI). Front. Hum. Neurosci. 11:423. doi: 10.3389/fnhum.2017.00423, PMID: 28936168 PMC5594081

[ref19] Dunlop K. Victoria L. W. Downar J. Gunning F. M. Liston C. (2021). Accelerated brain aging predicts impulsivity and symptom severity in depression. Neuropsychopharmacology 46, 911–919. doi: 10.1038/s41386-021-00967-x, PMID: 33495545 PMC8115107

[ref20] Elfwing S. Uchibe E. Doya K. (2018). Sigmoid-weighted linear units for neural network function approximation in reinforcement learning. Neural Networks 107, 3–11. doi: 10.1016/j.neunet.2017.12.012 29395652

[ref21] Elliott M. L. Belsky D. W. Knodt A. R. Ireland D. Melzer T. R. Poulton R.. (2021). Brain-age in midlife is associated with accelerated biological aging and cognitive decline in a longitudinal birth cohort. Mol. Psychiatry 26, 3829–3838. doi: 10.1038/s41380-019-0626-7, PMID: 31822815 PMC7282987

[ref22] Feng N. Geng X. Qin L. (2020). Study on MRI medical image segmentation technology based on CNN-CRF model. IEEE Access 8, 60505–60514. doi: 10.1109/access.2020.2982197

[ref23] Finn E. S. Constable R. T. (2016). Individual variation in functional brain connectivity: implications for personalized approaches to psychiatric disease. Dialogues Clin. Neurosci. 18, 277–287. doi: 10.31887/dcns.2016.18.3/efinn, PMID: 27757062 PMC5067145

[ref24] Fries P. (2015). Rhythms for cognition: communication through coherence. Neuron 88, 220–235. doi: 10.1016/j.neuron.2015.09.034, PMID: 26447583 PMC4605134

[ref25] Gaser C. Franke K. Klöppel S. Koutsouleris N. Sauer H. Initiative A. D. N. (2013). BrainAGE in mild cognitive impaired patients: predicting the conversion to Alzheimer’s disease. PLoS One 8:e67346. doi: 10.1371/journal.pone.0067346, PMID: 23826273 PMC3695013

[ref26] Gaudreau H. Carrier J. Montplaisir J. (2001). Age-related modifications of NREM sleep EEG: from childhood to middle age. J. Sleep Res. 10, 165–172. doi: 10.1046/j.1365-2869.2001.00252.x, PMID: 11696069

[ref27] Gratton G. Coles M. G. H. Donchin E. (1983). A new method for off-line removal of ocular artifact. Electroencephalogr. Clin. Neurophysiol. 55, 468–484. doi: 10.1016/0013-4694(83)90135-9, PMID: 6187540

[ref28] He C. Liu J. Zhu Y. Du W. (2021). Data augmentation for deep neural networks model in EEG classification task: a review. Front. Hum. Neurosci. 15:765525. doi: 10.3389/fnhum.2021.765525, PMID: 34975434 PMC8718399

[ref29] Herzberg M. P. McKenzie K. J. Hodel A. S. Hunt R. H. Mueller B. A. Gunnar M. R.. (2021). Accelerated maturation in functional connectivity following early life stress: circuit specific or broadly distributed? Dev. Cogn. Neurosci. 48:100922. doi: 10.1016/j.dcn.2021.100922, PMID: 33517108 PMC7848776

[ref30] Hesamian M. H. Jia W. He X. Kennedy P. (2019). Deep learning techniques for medical image segmentation: achievements and challenges. J. Digit. Imaging 32, 582–596. doi: 10.1007/s10278-019-00227-x, PMID: 31144149 PMC6646484

[ref31] Ioffe S. Szegedy C. (2015). “Batch normalization: Accelerating deep network training by reducing internal covariate shift” in International conference on machine learning. eds. Kingma D. P. Ba J. PMLR. 448–456. doi: 10.48550/arXiv.1502.03167

[ref32] Irmak E. (2021). Multi-classification of brain tumor MRI images using deep convolutional neural network with fully optimized framework. Iran. J. Sci. Technol. - Trans. Electr. Eng. 45, 1015–1036. doi: 10.1007/s40998-021-00426-9

[ref33] Jaworska N. Blier P. Fusee W. Knott V. (2012). Alpha power, alpha asymmetry and anterior cingulate cortex activity in depressed males and females. J. Psychiatr. Res. 46, 1483–1491. doi: 10.1016/j.jpsychires.2012.08.003, PMID: 22939462 PMC3463760

[ref34] Kaiser R. H. Andrews-Hanna J. R. Wager T. D. Pizzagalli D. A. (2015). Large-scale network dysfunction in major depressive disorder: a meta-analysis of resting-state functional connectivity. JAMA Psychiatry 72, 603–611. doi: 10.1001/jamapsychiatry.2015.0071, PMID: 25785575 PMC4456260

[ref35] Kingma D. P. Ba J. L. (2017). Adam: a method for stochastic optimization. Arxiv.

[ref36] Kolesnikov S. (2018). Catalyst: accelerated deep learning R&D. Available at: https://github.com/catalyst-team/catalyst (Accessed November 14, 2022).

[ref37] Leonardsen E. H. Peng H. Kaufmann T. Agartz I. Andreassen O. A. Celius E. G.. (2022). Deep neural networks learn general and clinically relevant representations of the ageing brain. NeuroImage 256:119210. doi: 10.1016/j.neuroimage.2022.119210, PMID: 35462035 PMC7614754

[ref38] Li X. La R. Wang Y. Hu B. Zhang X. (2020). A deep learning approach for mild depression recognition based on functional connectivity using electroencephalography. Front. Neurosci. 14:192. doi: 10.3389/fnins.2020.00192, PMID: 32300286 PMC7142271

[ref39] Madden D. J. Jain S. Monge Z. A. Cook A. D. Lee A. Huang H.. (2020). Influence of structural and functional brain connectivity on age-related differences in fluid cognition. Neurobiol. Aging 96, 205–222. doi: 10.1016/j.neurobiolaging.2020.09.010, PMID: 33038808 PMC7722190

[ref40] Marek S. Tervo-Clemmens B. Calabro F. J. Montez D. F. Kay B. P. Hatoum A. S.. (2022). Reproducible brain-wide association studies require thousands of individuals. Nature 603, 654–660. doi: 10.1038/s41586-022-04492-9, PMID: 35296861 PMC8991999

[ref41] Metzen D. Genç E. Getzmann S. Larra M. F. Wascher E. Ocklenburg S. (2022). Frontal and parietal EEG alpha asymmetry: a large-scale investigation of short-term reliability on distinct EEG systems. Brain Struct. Funct. 227, 725–740. doi: 10.1007/s00429-021-02399-1, PMID: 34676455 PMC8843903

[ref42] Montavon G. Lapuschkin S. Binder A. Samek W. Müller K.-R. (2017). Explaining nonlinear classification decisions with deep Taylor decomposition. Pattern Recogn. 65, 211–222. doi: 10.1016/j.patcog.2016.11.008

[ref43] Noor M. B. T. Zenia N. Z. Kaiser M. S. Mamun S. A. Mahmud M. (2020). Application of deep learning in detecting neurological disorders from magnetic resonance images: a survey on the detection of Alzheimer’s disease, Parkinson’s disease and schizophrenia. Brain Inform. 7:11. doi: 10.1186/s40708-020-00112-2, PMID: 33034769 PMC7547060

[ref44] Oh S. L. Vicnesh J. Ciaccio E. J. Yuvaraj R. Acharya U. R. (2019). Deep convolutional neural network model for automated diagnosis of schizophrenia using EEG signals. Appl. Sci. 9:2870. doi: 10.3390/app9142870

[ref45] Olah C. Mordvintsev A. Schubert L. (2017). Feature visualization. Distill 2. doi: 10.23915/distill.00007

[ref46] Rajkumar R. Farrher E. Mauler J. Sripad P. Brambilla C. R. Kops E. R.. (2021). Comparison of EEG microstates with resting state fMRI and FDG-PET measures in the default mode network via simultaneously recorded trimodal (PET/MR/EEG) data. Hum. Brain Mapp. 42, 4122–4133. doi: 10.1002/hbm.24429, PMID: 30367727 PMC8356993

[ref47] Rokicki J. Wolfers T. Nordhøy W. Tesli N. Quintana D. S. Alnæs D.. (2020). Multimodal imaging improves brain age prediction and reveals distinct abnormalities in patients with psychiatric and neurological disorders. Hum. Brain Mapp. 42, 1714–1726. doi: 10.1002/hbm.25323, PMID: 33340180 PMC7978139

[ref49] Schmaal L. Pozzi E. Ho T. C. van Velzen L. S. Veer I. M. Opel N.. (2020). ENIGMA MDD: seven years of global neuroimaging studies of major depression through worldwide data sharing. Transl. Psychiatry 10:172. doi: 10.1038/s41398-020-0842-6, PMID: 32472038 PMC7260219

[ref50] Sharma N. Jain V. Mishra A. (2018). An analysis of convolutional neural networks for image classification. Procedia Comput. Sci. 132, 377–384. doi: 10.1016/j.procs.2018.05.198

[ref51] Shorten C. Khoshgoftaar T. M. (2019). A survey on image data augmentation for deep learning. J. Big Data 6:60. doi: 10.1186/s40537-019-0197-0 PMC8287113 34306963

[ref52] Siman-Tov T. Bosak N. Sprecher E. Paz R. Eran A. Aharon-Peretz J.. (2017). Early age-related functional connectivity decline in high-order cognitive networks. Front. Aging Neurosci. 8:330. doi: 10.3389/fnagi.2016.00330, PMID: 28119599 PMC5223363

[ref53] Simonyan K. Vedaldi A. Zisserman A. (2013). Deep inside convolutional networks: Visualising image classification models and saliency maps. Arxiv. doi: 10.48550/arXiv.1312.6034

[ref54] Singh D. Kumar V. Yadav V. Kaur M. (2021). Deep neural network-based screening model for COVID-19-infected patients using chest X-ray images. Int. J. Pattern Recognit. 35:2151004. doi: 10.1142/s0218001421510046

[ref55] Smilkov D. Thorat N. Kim B. Viégas F. Wattenberg M. (2017). SmoothGrad: Removing noise by adding noise. Arxiv. doi: 10.48550/arXiv.1706.03825

[ref56] Springenberg J. T. Dosovitskiy A. Brox T. Riedmiller M. (2014). Striving for simplicity: The all convolutional net. Arxiv. doi: 10.48550/arXiv.1412.6806

[ref57] Sun J. Cao R. Zhou M. Hussain W. Wang B. Xue J.. (2021). A hybrid deep neural network for classification of schizophrenia using EEG data. Sci. Report 11:4706. doi: 10.1038/s41598-021-83350-6, PMID: 33633134 PMC7907145

[ref58] Sundararajan M. Taly A. Yan Q. (2017). “Axiomatic attribution for deep networks” in International conference on machine learning. PMLR. 3319–3328. doi: 10.48550/arXiv.1703.01365

[ref59] Sundaresan A. Penchina B. Cheong S. Grace V. Valero-Cabré A. Martel A. (2021). Evaluating deep learning EEG-based mental stress classification in adolescents with autism for breathing entrainment BCI. Brain Inform. 8:13. doi: 10.1186/s40708-021-00133-5, PMID: 34255197 PMC8276906

[ref60] Tang S. Wu Z. Cao H. Chen X. Wu G. Tan W.. (2022). Age-related decrease in default-mode network functional connectivity is accelerated in patients with major depressive disorder. Front. Aging Neurosci. 13:809853. doi: 10.3389/fnagi.2021.809853, PMID: 35082661 PMC8785895

[ref61] Van Den Heuvel M. P. Fornito A. (2014). Brain networks in schizophrenia. Neuropsychol. Rev., 24, 32–48. doi: 10.1007/s11065-014-9248-7 24500505

[ref62] Van Dijk H. Van Wingen G. Denys D. Olbrich S. Van Ruth R. Arns M. (2022). The two decades BrainClinics research archive for insights in neurophysiology (TD-BRAIN) database. Sci. Data 9:333. doi: 10.1038/s41597-022-01409-z, PMID: 35701407 PMC9198070

[ref63] Van Putten M. J. A. M. Olbrich S. Arns M. (2018). Predicting sex from brain rhythms with deep learning. Sci. Report 8:3069. doi: 10.1038/s41598-018-21495-7, PMID: 29449649 PMC5814426

[ref64] Vieira S. Pinaya W. H. L. Mechelli A. (2017). Using deep learning to investigate the neuroimaging correlates of psychiatric and neurological disorders: methods and applications. Neurosci. Biobehav. Rev. 74, 58–75. doi: 10.1016/j.neubiorev.2017.01.002, PMID: 28087243

[ref65] Wang J. Knol M. J. Tiulpin A. Dubost F. Bruijne M. De Vernooij M. W.. (2019). Gray matter age prediction as a biomarker for risk of dementia. Proc. National Sci. Acad., 116, 21213–21218. doi: 10.1073/pnas.1902376116, PMID: 31575746 PMC6800321

[ref66] Wong C. W. DeYoung P. N. Liu T. T. (2016). Differences in the resting-state fMRI global signal amplitude between the eyes open and eyes closed states are related to changes in EEG vigilance. NeuroImage 124, 24–31. doi: 10.1016/j.neuroimage.2015.08.053, PMID: 26327245

[ref67] Yamashita R. Nishio M. Do R. K. G. Togashi K. (2018). Convolutional neural networks: an overview and application in radiology. Insights Imaging 9, 611–629. doi: 10.1007/s13244-018-0639-9, PMID: 29934920 PMC6108980

[ref68] Zeiler M. D. Fergus R. (2013). Visualizing and understanding convolutional networks. Arxiv. doi: 10.48550/arXiv.1311.2901

[ref69] Zhang Y. Wu W. Toll R. T. Naparstek S. Maron-Katz A. Watts M.. (2020). Identification of psychiatric-disorder subtypes from functional-connectivity patterns in resting-state electroencephalography. Nat. Biomed. Eng. 5, 309–323. doi: 10.1038/s41551-020-00614-8 33077939 PMC8053667

[ref70] Zintgraf L. M. Cohen T. S. Adel T. Welling M. (2017). Visualizing deep neural network decisions: Prediction difference analysis. Arxiv. doi: 10.48550/arXiv.1702.04595

[ref71] Zoubi O. A. Wong C. K. Kuplicki R. T. Yeh H. Mayeli A. Refai H.. (2018). Predicting age from brain EEG signals—a machine learning approach. Front. Aging Neurosci. 10:184. doi: 10.3389/fnagi.2018.00184, PMID: 30013472 PMC6036180

