# Supplementary material for: Predicting age from resting-state scalp EEG signals with deep convolutional neural networks on TD-brain dataset
Source: Front Aging Neurosci. 2022 Dec 6;14:1019869. doi: 10.3389/fnagi.2022.1019869 (PMC9764861; doi:10.3389/fnagi.2022.1019869)
Supplement: Supplementary file 1 [file Data_Sheet_1.PDF]

# Supplementary materials

## Choosing sessions for the DCNN analysis

The TD-Brain dataset consists of 1,274 patients with a total of 1346 sessions. For 69 participants more than one session recorded at different times are available (two sessions for 65 participants and three sessions for 4 participants). The distribution of time intervals between the sessions from participants with two sessions are presented in Supplementary Figure 1.

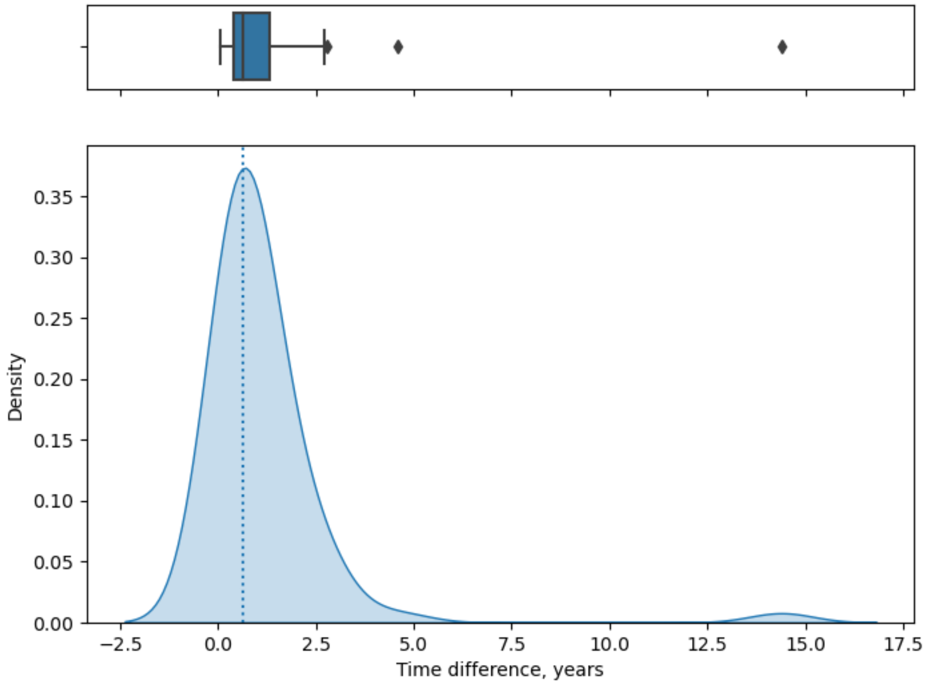


**SUPPLEMENTARY FIGURE 1 |** The distribution and box plot of the time intervals between recording sessions. The dashed line represents the median of the distribution.

The time interval between the repeated sessions is ranged from 2 months to 14 years with the mean interval 1.16 year (median interval 0.63 years).

## The age-related distribution of the brain disorders

The TD-Brain is a heterogeneous dataset with both healthy participants and participants with different types of disorders. For the purpose of age prediction, the uneven and correlated with the age distribution of disorders can create additional information that the DCNN model can use for training. In the initial dataset there are two variables describing the status of the participants - formal disorder ("formal.Dx" variable) and the status measured by different types of methods, including, for example, specific questionnaires (the "indication" variable). The details can be find in Dijk et al., 2022). To analyze the potential effects of the uneven distribution of the different types of disorders we created the merged variable ("merged_indications") that included information from both "formal.Dx" and "indication" variables.

For the purpose of the present analysis the different types of indicators were combined into 13 classes. The indication types with fewer than 10 participants were combined into the type "Other". The number of participants in different groups substantially differed (see Supplementary Table 1).

| Merged_indication type | Sample size, $N$ |
|---|---|

| | |
|---|---|
| MDD | 389 |
| UNKNOWN | 253 |
| ADHD | 236 |
| SMC | 117 |
| OTHER | 98 |
| OCD | 65 |
| HEALTHY | 46 |
| INSOMNIA | 32 |
| TINNITUS | 30 |
| PARKINSON | 26 |
| Dyslexia | 19 |
| CHRONIC PAIN | 14 |
| BURNOUT | 1 |

**SUPLLEMENTARY TABLE 1 |** The number of participants in different “merged_indication” groups.

The age distributions for the participants with different types of disorders are presented in Supplementary Figure 2.

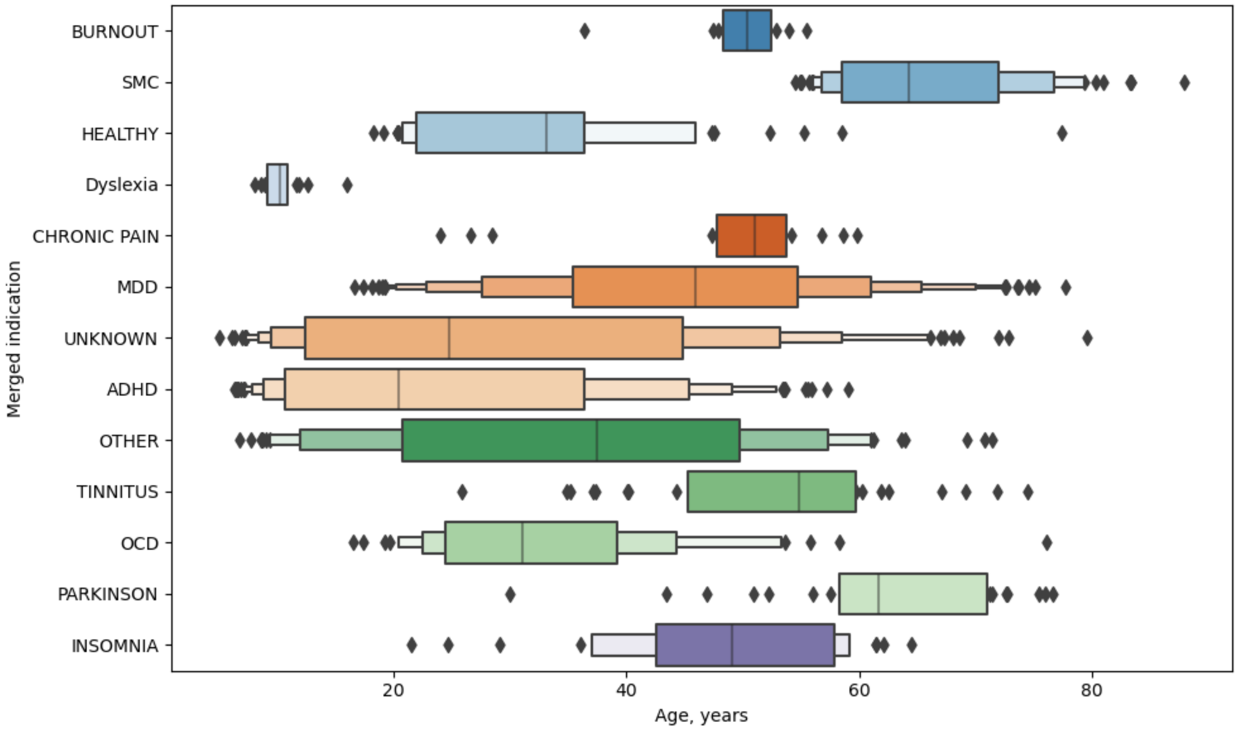


**SUPPLEMENTARY FIGURE 2 |** The age distribution for different types of disorders.

Although in one dataset it is impossible to completely disentangle age per se and other factors correlated with age, we have developed a multiclass prediction model to estimate the accuracy of the prediction of different types of disorders. Overall, the prediction accuracy of the multiclass model was low. The weighted average prediction accuracy was 39% (Supplementary Table 2). The confusion matrix for the model is presented in Supplementary Figure 3.

| | **Precision** | **Recall** | **f1-score** | **support** |
|---|---|---|---|---|
| BURNOUT | 0.46 | 0.47 | 0.46 | 236 |
| SMC | 0.00 | 0.00 | 0.00 | 10 |
| HEALTHY | 0.00 | 0.00 | 0.00 | 14 |
| Dyslexia | 0.34 | 0.58 | 0.43 | 19 |
| CHRONIC PAIN | 0.31 | 0.48 | 0.38 | 46 |
| MDD | 0.22 | 0.50 | 0.30 | 32 |
| UNKNOWN | 0.53 | 0.35 | 0.42 | 389 |
| ADHD | 0.20 | 0.40 | 0.27 | 65 |
| OTHER | 0.15 | 0.19 | 0.17 | 98 |
| TINNITUS | 0.19 | 0.42 | 0.26 | 26 |
| OCD | 0.71 | 0.83 | 0.76 | 117 |
| PARKINSON | 0.27 | 0.27 | 0.27 | 30 |
| INSOMNIA | 0.42 | 0.27 | 0.33 | 253 |
| | | | | |
| accuracy | | | 0.39 | 1335 |
| macro avg | 0.29 | 0.37 | 0.31 | 1335 |

| | | | | |
|---|---|---|---|---|
| weighted avg | 0.43 | 0.39 | 0.40 | 1335 |

**SUPLLEMENTARY TABLE 2 |** The prediction accuracy of the DCNN model for different "merged_indication" groups.

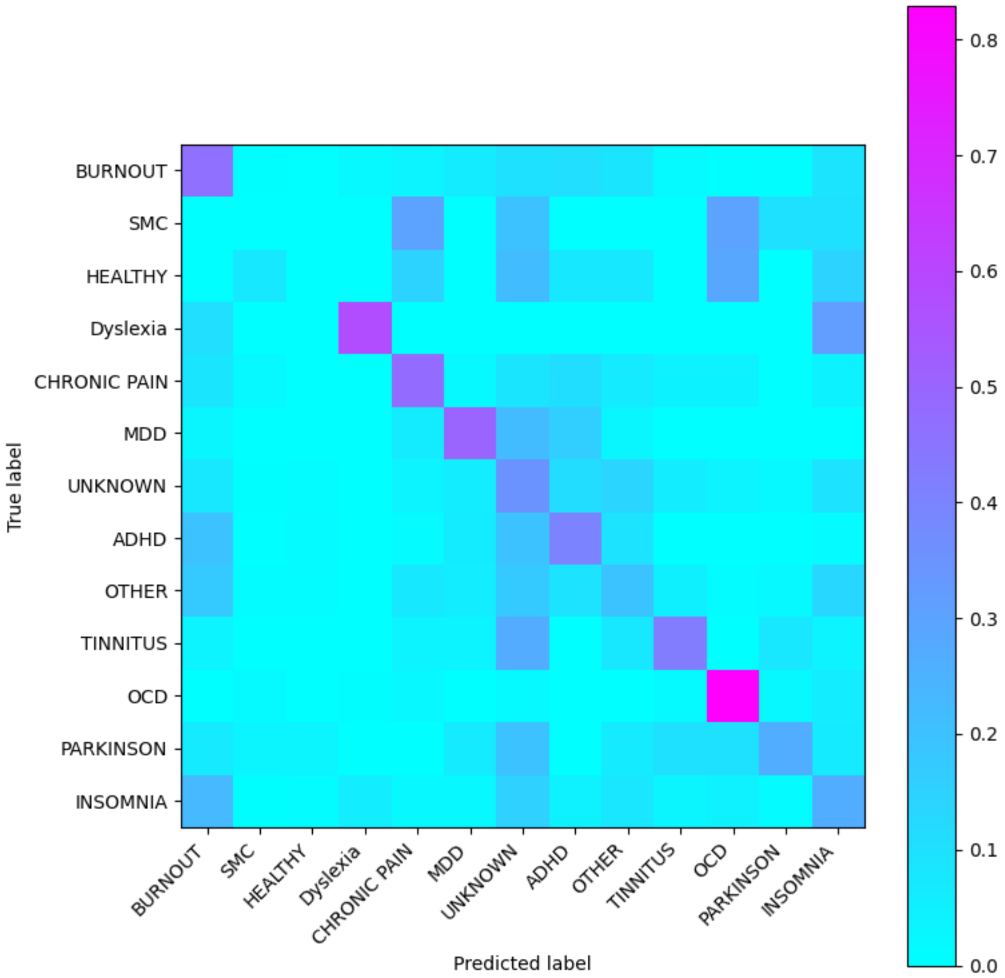


**SUPPLEMENTARY FIGURE 3 |** The confusion matrix for the multiclass "merged_indication" DCNN prediction model.
